# SMART: A Spatially Explicit Bio-Economic Model for Assessing and Managing Demersal Fisheries, with an Application to Italian Trawlers in the Strait of Sicily

**DOI:** 10.1371/journal.pone.0086222

**Published:** 2014-01-23

**Authors:** Tommaso Russo, Antonio Parisi, Germana Garofalo, Michele Gristina, Stefano Cataudella, Fabio Fiorentino

**Affiliations:** 1 Laboratory of Experimental Ecology and Aquaculture, Department of Biology, “Tor Vergata” University of Rome, via della Ricerca Scientifica s.n.c., Rome, Italy; 2 Department of Economics and Finance, Faculty of Economics, “Tor Vergata” University of Rome, Rome, Italy; 3 National Research Council (CNR), Institute for Coastal Marine Environment (IAMC), Mazara del Vallo, Italy; Technical University of Denmark, Denmark

## Abstract

Management of catches, effort and exploitation pattern are considered the most effective measures to control fishing mortality and ultimately ensure productivity and sustainability of fisheries. Despite the growing concerns about the spatial dimension of fisheries, the distribution of resources and fishing effort in space is seldom considered in assessment and management processes. Here we propose SMART (Spatial MAnagement of demersal Resources for Trawl fisheries), a tool for assessing bio-economic feedback in different management scenarios. SMART combines information from different tasks gathered within the European Data Collection Framework on fisheries and is composed of: 1) spatial models of fishing effort, environmental characteristics and distribution of demersal resources; 2) an Artificial Neural Network which captures the relationships among these aspects in a spatially explicit way and uses them to predict resources abundances; 3) a deterministic module which analyzes the size structure of catches and the associated revenues, according to different spatially-based management scenarios. SMART is applied to demersal fishery in the Strait of Sicily, one of the most productive fisheries of the Mediterranean Sea. Three of the main target species are used as proxies for the whole range exploited by trawlers. After training, SMART is used to evaluate different management scenarios, including spatial closures, using a simulation approach that mimics the recent exploitation patterns. Results evidence good model performance, with a noteworthy coherence and reliability of outputs for the different components. Among others, the main finding is that a partial improvement in resource conditions can be achieved by means of nursery closures, even if the overall fishing effort in the area remains stable. Accordingly, a series of strategically designed areas of trawling closures could significantly improve the resource conditions of demersal fisheries in the Strait of Sicily, also supporting sustainable economic returns for fishermen if not applied simultaneously for different species.

## Introduction

Demersal assemblages exploited in multi-species fishery are characterized by a wide array of species, each having a distinct population dynamic and pattern of occurrence in space and time. Also fishing activity has its spatial and temporal dynamic. Fishing effort distribution is driven by fishermen's knowledge of spatio-temporal distribution and abundance of resources which, in turn, depend upon abiotic and biotic environment features, ontogenetic and/or seasonal movements, and fishing efforts. In addition, fishermen spread their efforts over different areas on the basis of a series of *a priori* evaluations and an *a posteriori* feedback of costs and catches, where one of the main pay-offs is represented by the maximization of profits [1]. The interaction between spatial dynamics of biological resources and fishing behavior frequently generates complex spatial patterns of fishing mortalities on exploited stock [2]. In the last decade, amid growing concerns for the ecological aspects of fishery science, the regulation of fishery activities in areas critical for the life cycle of commercial species, such as nursery and spawning grounds, and in sensitive habitats, such as sea grass beds and maerl communities, is increasingly advocated as a complementary tool in conventional fishery management [3]. Thus, assessment and management of fisheries are moving toward a class of spatially explicit and bio-economic approaches in which 1) both the impact of fishing activities and the response of resources in space are taken into consideration and possibly modeled, and 2) management measures are evaluated on the basis of their observed or hypothesized effects in space [4]–[7]. In this regard, the assessment of the impact of fishing activity (and therefore the expected effects of alternative management scenarios) is a crucial point to be investigated, and may ultimately be identified in the spatially-resolved fishing mortality of resources. The absence of an adequate knowledge of fishermen's dynamics (rather than of stock biology) has been identified as the main critical aspect of fishery management [8]. Nevertheless, there is still a lack of effective tools for predicting the biological and economic effects of different scenarios of effort allocation in mixed fisheries, so that the management of mixed fisheries has generally been unsuccessful in the various different contexts [9].

This paper proposes a spatial model for assessing the state of demersal resources and certain aspects of bio-economic performance under different management scenarios. Our model, called SMART (Spatial MAnagement of demersal Resources for Trawl fisheries), combines information from experimental trawl surveys, monitoring of commercial catches and remote sensing of vessel activity (captured from data provided by the Vessel Monitoring System - VMS), gathered within the European Data Collection Framework on fisheries (EC Regulation no 199/2008). The SMART model is applied to assess behavior of demersal fisheries in the Strait of Sicily, which represents one of the most productive fisheries of the Mediterranean Sea [10], [11]. Within a complex of demersal species exploited in the area, the deep water rose shrimp (DPS – *Parapenaeus longirostris*, Lucas 1847), the European hake (HKE – *Merluccius merluccius*, Linnaeus 1758) and the red mullet (MUT – *Mullus barbatus*, Linnaeus 1758), which account for about 54% of total yield and 70% of total revenue from groundfish resources of the Strait of Sicily, have been selected as target species in the present study.

The basic idea of our model arises from the following assumptions: (1) the mean pattern of distribution of demersal resources is influenced by sea bottom characteristics and mean annual sea surface temperature, which should represent two of the main environmental variables affecting the spatial pattern and productivity of benthic communities [11], [12]; (2) resource abundance in space and its variations on a short temporal scale (from one year to the next) are driven by the combined effect of internal (demography) and external (fishery) factors [13], [14]; (3) in mixed fisheries, each fisherman independently and freely exploits different resources in order to compose a selection based on a series of constraints imposed by economic factors (e.g., market prices, costs) which can change over time.

SMART was developed by setting up and combining the following four tools:

1. A spatial analysis approach which models the distribution of demersal resources, fishing effort and abiotic factors in order to produce matrices of geo-referenced data in the investigated area for the years 2006–2010;
2. An Artificial Neural Network (ANN) which captures the relationships between resources, fishing effort and abiotic factors on the basis of the time series of matrices obtained from the previous step, and then predicts resources abundance and distribution in the near future;
3. A deterministic model that computes the specific size structure of catches corresponding to a given combination of resources distribution and fishing effort using classic fishery science equations. These catches are then converted into revenues on the basis of market prices by species/size, while a simple model is used to compute the fuel costs associated to the fishing effort pattern. Finally, revenues and costs are used to obtain gains;
4. A simulation approach using the previous tools to explore the effects of different management scenarios of fishing effort on resources abundance in the near future. This component of the model works by iteratively generating patterns of fishing effort for different scenarios and then applying tools 2 and 3 to predict the bio-economic effects.

Our approach is effective in generating patterns of fishing effort, which are similar to the current ones and/or satisfy some constraints imposed by the manager (e.g. the displacement of a fraction of effort from one area to another). The findings provide important indications about the effects of fishing mortality changes, even as result of partitioning and managing fishing mortality in space. Moreover, SMART could potentially be a tool in the context of Integrated Ecosystem Assessments and Integrated Ecosystem-Based Management, since it integrates the interactions among the different components of the system (including the anthropogenic one), while it uses and valorizes a large platform of data mandatorily collected in EU seas and shared among researchers of member states.

## Materials and Methods

### The Strait of Sicily and its fisheries

**Figure 1:**
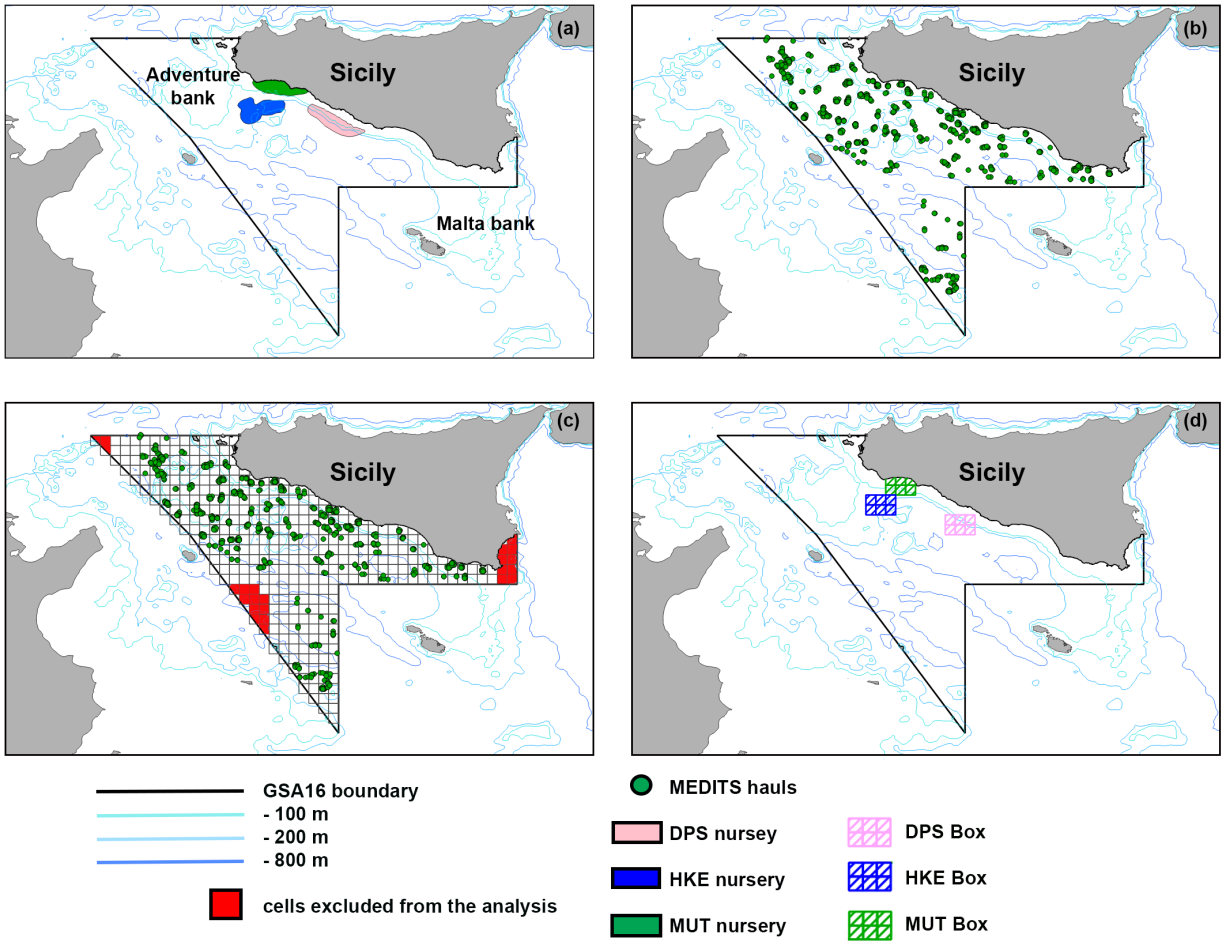
a) Map of the GSA16 (Strait of Sicily), with bathymetry represented by level line and nursery areas identified by [62]; b) positions (green points) of the Mediterranean International Trawl Survey (MEDITS) hauls, which are the sample design annually repeated during the survey; c) the grid (394 cells of size 6 min×6 min) used in the present study. Green points allows to identify cells containing MEDITS hauls, while cells not interpolated and excluded from the analysis are filled in red; d) Position of the three proposed boxes (overlapping nurseries) for fishing closure.

The study area comprises the Italian side of the Strait of Sicily (SoS) (Fig. 1a), that is the portion of Mediterranean sea identified as Geographic Sub Area (GSA) 16 [15]. The area covers about 34,000 km^2^ with a wide range of water depths and habitats due to the complexity of the bottom morphology. Along the Sicilian coast, the shelf is characterized by two wide and shallow banks (<100 m depth) in the western (Adventure Bank) and eastern (Malta Bank) sectors respectively, separated by a narrow shelf in the middle. SoS is one of the most important fishing areas for demersal resources in the Mediterranean in view of the large fleets operating there and the relative fish production [16], [17]. Sicilian trawlers between 12 and 24 m LOA are based in seven harbors along the southern coasts of Sicily. These trawlers operate mainly in the form of short-distance fishing trips ranging from 1 to 2 days at sea, and fishing takes place on the outer shelf and upper slope. Sicilian trawlers measuring over 24 m in LOA go on longer fishing trips, which may last up to 4 weeks. These vessels operate offshore, in both the Italian and international waters of the Strait of Sicily [17] (Fig. 1a).

During 2009–2011 the mean total yield of demersal species was 17753 tons (sd = 546) [18]. At present, deep-water pink shrimp (*P. longirostris* - DPS) is the main target species, accounting for about 41.7% of the demersal yield, with European hake (*M. merluccius* - HKE) and red mullet (*M. barbatus* – MUT) totaling 8.2 and 4.0% of the landing, respectively. According to the more recent assessments carried out inside the Scientific Advisory Committee - General Fisheries Commission for the Mediterranean (SAC-GFCM) [19] and the Subgroup on the Mediterranean - Scientific, Technical and Economic Committee for Fisheries (SGMED STECF) [20], the status of all three species is “overfished”. Based on Length Cohort Analysis and Yield per Recruit Analysis, the current fishing mortality (F) would have to be reduced by values ranging from 20% (DPS) to 60% (HKE) in order to reach a more sustainable exploitation in the area.

DPS is the main target of demersal trawling in the area and it is fished exclusively by trawling in the outer shelf upper slope. A strong relationship between size and depth has been observed, with the smallest and youngest specimens inhabiting shallower waters [21].

HKE is a necto-benthonic fish living at depths of between 10 and 1000 m, although it is found mostly between about 70 and 400 m. The bathymetric distribution of this species is related to size, the smaller specimens being caught more frequently on the outer continental shelf (50–200 m depth), while the larger ones are mainly distributed along the continental slope [22]. The majority of hake catches (more than 95%) is obtained by bottom trawling, although the species is fished also using longlines and gillnets [23].

MUT is a benthic species, frequently found on muddy bottoms at depths of between 5 and 250 m [24]. In SoS, it is fished almost exclusively by bottom trawlers [25].

### Abundance and spatial distribution of target species

Geo-referenced abundance data of target species, collected during the “Mediterranean international bottom trawl survey” (MEDITS) program, were processed in order to obtain the quantitative spatial distributions for the years 2006–2010. The MEDITS survey program is designed to produce basic information on demersal species in terms of abundance and demographic structure as well as spatial distribution [26], [27]. MEDITS has been carried out annually in spring/summer since 1994. Sampling stations are replicated each year and selected using a stratified random sampling design based on five depth strata: 10–50 m, 51–100 m, 101–200 m, 200–500 m, 500–800 m, where the number of hauls is proportional to the area of each stratum (Fig 1b). The surveys were carried out using the same vessel, equipment and protocol throughout the entire period [26]. Catches by haul were processed, sorted to species level, and weighed as total catch. Sex, maturity stage and length of each specimen were also collected. The procedure extensively described in [26] and [28] was applied to compute abundances by species/size class, standardized to 1 km^2^ (N/km^2^), in each bathymetric stratum. In this way, the total abundance *N* of individuals of the species *s*, for the size class *l*, in the year *t*, is equal to
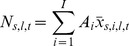
where *A_i_* is the area of stratum *i*, *I* is the total number of strata, and 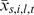 is the mean number of individuals (specimens per km^2^) of the size class *l*, at year *t*, in the hauls relative to stratum *i*.

These abundance indices were then used to reconstruct the specific length-frequency distribution (LFDs) for each year. The LFDs obtained in this way were inspected in order to identify the optimal number of normal components (cohorts) for each year and their characteristics in terms of proportion of total abundance, mean length (in mm) and standard deviation. This step was performed using the mixture analysis provided in R [29] by the package mixdist [30] and offered the advantage of drastically reducing the number of variables needed to characterize the LFD in the grid cells. Given that the relationship between length and age is different for the two sexes in all three species [31], data were processed keeping the sexes separate. Due to the weak correspondence between length and age in the largest size of the LFDs, the number of components for each sex/species/year LFDs was set as three for DPS and HKE (the latter grouping all the specimens of age group 3+ years) and two for MUT. The mixture analysis produced a set of overlapping component distributions that provides the best fit for grouped data and conditional data using a combination of a Newton-type method and EM algorithm provided by mixdist [30]. The goodness-of-fit of these outputs was assessed by comparing the quantiles of observed and fitted distributions from the distance of Kolmogorov-Smirnov (K-S test). [32]–[34]. For exploratory purposes, the mixtures for each species were converted into biomass distribution using specific length-weight relationships (Table 1).

**Table 1:** Parameters used for the different modules of SMART [22]–[25], [90]–[92].

*Code*	*Parameter*	*P. longirostris (*♂*)*	*P. longirostris (*♀*)*	*M. merluccius (*♂*)*	*M. merluccius (*♀*)*	*M. barbatus (*♂*)*	*M. barbatus (*♀*)*
***L_∞_***	Length at infinite age	33.56	42.71	1000	201.6	236.1	33.56
***k***	Growth rate	0.73	0.67	0.116	0.57	0.45	0.73
***t_0_***	Time at which length is zero	−0.13	−0.208	−0.5	−0.8	−0.8	−0.13
***M***	Natural mortality	1.2	1.05	0.43	0.34	1.0	1.0
**a**	Length-Weight parameter	0.0034	0.0029	0.000003598	0.000003598	0.00002648	0.00001532
**b**	Length-Weight parameter	2.4096	2.4818	3.125	3.125	2.823	2.942
***L_50_***	Selectivity of trawl net (40 mm)	15	15	120	120	90	90
***L_75_***	Selectivity of trawl net (40 mm)	18	18	140	140	110	110
***q_s_***	Catchability of trawl net	0.0022	0.0022	0.0018	0.0018	0.0021	0.0021
***qM_s_***	Catchability of MEDITS net	0.85	0.85	0.15	0.15	0.85	0.85
***F (from LCA)***	Mean ± sd for the total fishing mortality in the years 2006–2010	1.33±0.16	1.33±0.16	1.15±0.12	1.15±0.12	1.47±0.17	1.47±0.17

After proportions by size in different years were identified in the cohorts, the absolute abundance of individuals belonging to each cohort was computed for each sampling station (Fig. 1b), based on observed LFDs. Hence, the annual spatial distribution of cohort abundance of each species was generated by inverse distance-weighted deterministic interpolation [35], which has been preferred to other approaches (e.g. kriging) since it does not require strong assumptions such as isotropy. A grid cell of 6 min×6 min (394 cells - Fig. 1c) was adopted, corresponding to a subdivision of the 30 min×30 min grid coded by the General Fisheries Commission for the Mediterranean [36]. The cell size was chosen taking into account the spatial resolution of the MEDITS sampling, as the median distance among hauls was about 4 nautical miles. The interpolation procedure was carried out considering a neighborhood with a radius of 3 cells. The cells for which no sampling station was available within the fixed radius were excluded from the interpolation (Fig. 1c) as well as from the subsequent analysis steps.

The final output of this analysis is represented by a series of matrices, one for each/species/year. The matrix rows represent the cells of the maps, while the columns refer to the cohorts (male and female summed), and values are the estimated abundances. These matrices were used as input for the subsequent analyses.

### Abiotic factors

Sea bottom depth was considered as a proxy for the environmental characteristics affecting demersal resources distribution. This assumption is largely supported in literature [11], [12], [37], [38]. A digital bathymetry grid at 1 min resolution was obtained using the Geodas Grid Translator of the National Geophysical Data Center (NOAA – available online at: www.ngdc.noaa.gov/mgg/global/global.html). Given that the map unit (cell) used in this study was 6 min×6 min, 36 depth pixels were associated to each cell. These pixels were used to compute two indexes: the first one (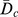) was the mean depth within each cell. The second one (*Dsd_c_*) was the standard deviation of the depth, and it was used as a proxy of the heterogeneity of the sea bottom within each cell. Moreover, the mean annual sea surface temperature (*SST*) was computed from the daily data available at the National Oceanographic Data Center (NODC - http://data.nodc.noaa.gov/) and processed to obtain annual values for each cell of the grid.

### Spatial distribution and intensity of the fishing effort

Fishing effort was used to quantify resources exploitation (fishing mortality) as a function of fishing gear (trawl) deployed in each cell of the spatial grid. It was estimated using data collected by the Vessel Monitoring System (VMS), introduced in 2002 by the European Union [39] for the remote control of fishing vessels and collected within DCF since 2006. Specifically, VMS data for the years 2006–2010 from the activity of about 300 vessels (the number slightly varied among different years) operating in the SoS (Fig. 1a) were used. The VMS data were processed following the methodology described in [40]–[42]. Briefly, single fishing tracks (series of trips of a fishing vessel, starting and ending in a given harbor) were cleaned and interpolated at 10-minute time intervals in order to obtain standardized records of vessel positions; then a speed filter was used to distinguish between fishing and non-fishing pings; finally, records of vessel positions in a fishing state (fishing points) were counted within each grid cell. The number of fishing points was assumed to be a reliable proxy of fishing effort, the spatial distribution of which was produced at the resolution of the 6 min grid.

### Predictive model of resources abundances: Elman artificial neural network

**Figure 2:**
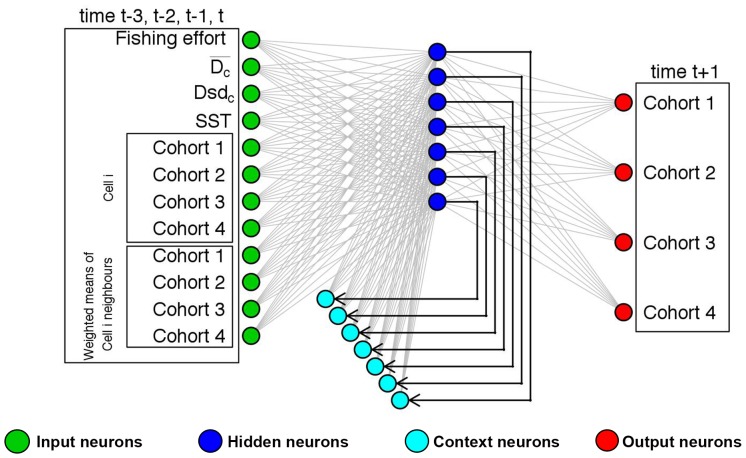
Representation of the Elman network used in this study for the *Mullus barbatus* (MUT) model. The input layer comprises: four neurons related to cohort abundances (two cohorts for males and two cohorts for females) at each time for each cell; other four neurons related to abundance of the same cohorts in the neighboring cells (ray = 2); a neuron for the fishing effort; three neurons for the sea bottom characteristics and mean annual sea surface temperature. These neurons directly propagate the information to the basic hidden neurons. At each step of the training procedure, the updated pattern of the basic hidden neurons is memorized by the context neurons and, at the successive step, propagated to the basic hidden neurons together with the new information in the input neurons. The output layer contains as many neurons as the number of cohorts in the input layer.

The spatial distribution of the target species is assumed to be a function of environmental factors (abiotic factors), specific demography (biotic factors) and fishing effort (anthropogenic factors), the latter representing an external source of size-dependent mortality. In practice, we attempted to predict abundance and spatial distribution of target species at year *t+1* on the basis of the following predictors: (1) gridded indices of sea bottom morphology and *SST* to summarize abiotic factors, (2) gridded indices of abundance by size class of target species from the previous *n* years, recorded from mid-year surveys to approximate the mean annual spatial distribution of the resources, (3) gridded indices of fishing effort, originated by VMS, from the previous *n* years. In this way *A_s,k,h,t+1,c_*, that is the abundance (in terms of number of individuals) of the cohort *h*, for the sex *k* of the species *s*, at year *t+1* in the cell *c*, is given by an unknown function *f*:
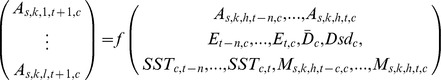
where *A_s,k,h,t-n,c_* is the species abundance and *E_s,h,t-n_* is the fishing effort at year *t-n*, respectively, while *SST_c,t_* is the sea surface temperature of cell *c* at year *t* and *M_s,k,h,t_* is the mean abundances for the same species/sex/cohorts in the neighboring cells of *c* (ray equals to two) at year *t*. Instead of attempting to model this complex relationship via an explicit quantitative panel such as a system of equations, an Elman multilayer perceptron network (EMPN) was used since it is the simplest and most widely used ANN architecture to pursue classification issues when sequential or time-varying patterns are inspected [43]. The basic structure of a multilayer perceptron network (MPN) consists of at least three layers of neurons (also called units or nodes – Fig. 2). Information flows in a unidirectional way from the first (input) layer to the last (output) layer through a second (hidden) layer and is processed in parallel by the neurons of each layer. The input layer contains as many neurons as there are independent variables or descriptors used to predict the dependent variables, which in turn constitute the output layer. Neurons of a given layer are linked to the neurons of the next layer by activation functions: hidden layer neurons compute a weighted sum of the input variables through a first activation function and then send a result to the output neurons through a second activation function. A sigmoid function, which is the most common choice because it is non linear and characterized by a very easy to compute derivative [44], [45], was chosen in both cases.

The EMPN was devised to predict the absolute abundances (Log_10_ of number of individuals) of each cohort of each species, for each cell at year *t* (2010), using the relative abundances provided by the MEDITS survey. The input variables are the abundances of the same cohorts for the previous four years (*t-1*,…, *t-4*, that is 2006–2009), the physical characteristics for the cell and the observed values of fishing effort for the same years. Conversely, it is possible to observe (Fig. 2) that the hidden layer of an EMPN contains the so called context neurons in addition to the common “basic” hidden neurons (it was preferred to adopt the adjective “basic” in order to avoid the confusion generated by the fact that the term “hidden” is used to define both the whole layer and a subgroup of neurons). In effect, Elman MPN (among others) is a class of ANN explicitly devised to process data organized on time series, and this is the reason for the presence of context neurons. These neurons are used to memorize the previous activations of the hidden neurons and can be considered as one-step time delays. Although there are no technical reasons for restricting the prediction to a short time horizon, it is generally accepted (as suggested by the common sense) that prediction should be restricted to a temporal depth no smaller than that of the time series used for training [44], [46]. For this reason we devised the EMPN to perform prediction for just one year. More details about EMPN training, which is analogous to the more classic MPN set up, can be found in Appendix S1 [47]–[51]. Given that EMPN training is sensitive to a random initialization step, the EMPN for each species was trained 100 times and the results was inspected before selection of the EMPNs to be used in the successive analyses. The performances of the trained EMPNs were summarized by the Pearson' correlation coefficient between the matrix of the observed and predicted standardized abundances. At the end of this procedure, the EMPN showing the best performance was selected (between the 100 trained) for each species. The EMPN used in this study was implemented in R [29] using the RSNNS package [52].

The relative importance of each input variable is a critical aspect when using ANN. Given that each input variable is associated with a set of weights (one for each neuron in the hidden layers), the relative importance of input variables is intuitively linked to the values of weights for the trained MPN [44] which ultimately represent a series of coefficients for an input variable. Thus, the effect changes of an input variable are linked to the value of its corresponding weights in the hidden layers. However, this offers the opportunity to evaluate the relative contribution of each input variable (or group of variables) to the obtained predictions. In this study, the patterns of weights in the hidden layers were explored, following the rationale proposed by [53] and applied in [41], to assess the influence of each group of input variables as relative importance (RI). RI ranges between 0 and 100 with a cumulative sum of 100.

### Bio-economic model for catches, revenues and gains: the spatial-partitioning of *F*

A set of quantitative relationships was used to build a deterministic model of catches and related revenues corresponding to a given combination of resource distributions and fishing effort in space and time. The model analytically relates abundances of resources by length to fishing effort for each cell of the grid, so that the specific length structure of catches can be determined. Then, the cumulative length structure of catches was obtained for each species by summing all the distributions obtained for all cells of the grid. These specific catches were then converted into specific revenues using the market prices by size category recorded for each year and, finally, total revenues were computed by summing the specific revenues of the three species.

Underpinning this process is the spatial-partitioning of the overall fishing mortality by species (*F_s_*), i.e. the data on fishing effort and resource abundances are used to resolve *F_s_* into its components (*f_s,c_*) for each species/cell.

The first step of the analysis involves the computation of spatial fishing mortality *f_s,c,l,t_* as [54]:
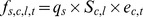
Where 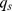 is the catchability coefficient of the commercial fleet for all the size class of species *s*, *S_s,l_* is the selectivity of trawl for the *l-th* length class of the species s, while *e_c,t_* is the effort deployed in cell *c* at year *t*. Notice that, in this way, it is assumed that all the vessels of the fleet are characterized by the same catching power. Future investigation on this topic could allow overcoming this assumption.

The selectivity of the trawl can be modeled by the “explicit” logistic curve [55]:
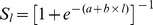
Here S*_l_* represents the proportion of the specimens retained within the codend in length class *l*, *a* is the steepness parameter:
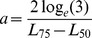
with *L_75_* and *L_50_* corresponding to size at capture of 75% and 25%, respectively, and *L_50_* denoting the size at capture of 50%. The selectivity parameters for a mesh size of 40 mm and 50 mm diamond opening in the cod end were obtained from literature (see Table 1).

The catchability coefficient *q_s_* of the commercial fleet was estimated for each species by using as reference the overall specific fishing mortality (*F_s_*) provided by Length Cohort Analysis (LCA) [19], [20]. More in detail, for each year in the period 2006–2010, we estimated the amount of harvested individuals in each cell as the product of the *F_s_* by the mean density in number of individuals in the cell, obtaining a vector (a value for each cell) of theoretical catches. Then the values of *q_s_* which minimizes the vectorial difference between these catches and those computed multiplying the mean density in number of individuals in the cell *c* (*q_s_*×*e_c_*) - with *e_c_* being being the fishing effort in cell c) was searched via the optimization algorithm provided by the R basic function “optim”.

Then, the catches by size were estimated as:
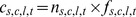
where *c_s,c,l_*, is the catch for the *l*-th length class of the species *s* in cell *c* at year *t, n_s,c,l,t_* represents the mean abundance for the *l-th* length class of a given species *s* in cell c at year *t*, approximated using the MEDITS abundance indices and *f_s,c,l,t_* is the harvest rate computed as described in section 3.5.

Given that the indices by cell obtained by MEDITS were expressed as relative indices, they were multiplied by the catchability coefficient by species (*qM_s_*) of MEDITS gear in order to obtain absolute estimates of standing stock by cell. This coefficient was calculated as the ratio between the absolute standing stock in number in GSA 16 from LCA (*N_LCA_*) [19]–[21] and the relative standing stock in number obtained from the swept area method (*N_swept area_*) using the MEDITS catch rates over the whole GSA16 surface:
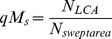
The harvested biomass for each species, cell, length class and year (*h_s,c,l,t_*) was calculated by multiplying the numbers of individuals caught *c_s,c,l,t_* by their length-specific average weights *w_s,c,l,t_* estimated from the length-at-age function and the length–weight relationship (parameters for these functions are reported in Table 1):
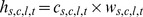
The total revenue of the three aggregated species for each year *R_t_* was calculated as follows:
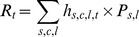
where *P_s,l_* are the average prices by size in 2006–2010 for the three species landed by trawlers in the main ports of the Strait of Sicily (Mazara del Vallo and Sciacca) (see Table 2).

**Table 2:** Prices for different ranges of size, for the three species (source: National Research Council, Institute for coastal marine environment).

DPS (*P. longirostris*)	
Size (mm)	Prices(Euros/kg)
<21	2.5
[21–25)	5
[25–30)	8
> = 30	16

HKE (*M. merluccius*)	
Size (mm)	Prices(Euros/kg)
<300	<300
> = 300	> = 300

MUT (*M. barbatus*)	
Size (mm)	Prices(Euros/kg)
<160	<160
> = 160	> = 160

For control purposes, the values of Biomasses, Catches and Total Fishing Mortality computed by SMART for year 2010 were compared to those available in literature [22]–[25] and online [18].

Fishing costs are generally distinguished between operational costs (i.e. variable/running costs and fixed costs) that are important in analyzing short-term economic performance and capital costs [56], [57]. A simple model was used in this study to estimate the operational costs (i.e. fuel cost) associated to each real or simulated pattern of fishing effort. For the trawler fleet operating in the SoS, these costs account for over the 80% of the total costs sustained by fisherman [18], and then can be considered as a good proxy of the current whole cost value for this fleet. The model assumes that the cost associated to a given fishing effort pattern is a linear function of: 1) the mean annual price of fuel (*E_t_*); and 2) a Pattern Score (*PS_t_*) measure related to the fishing zone, which captures the distance at which fishing operations are carried out. This rationale is very similar to the one applied in [57]. These authors, while evidencing a good fit for their data, also reported a strong effect of fuel price and fishing effort on the final costs sustained by each vessel. Here we compute the *PS_t_* as the yearly cumulative sum of the product of the fishing effort recorded in each cell (as Log10 of the number of fishing set positions) by the mean distance of the same cell from the three nearest harbors. The yearly total fuel cost for the fleet was regressed on the Pattern Score together with the mean annual cost of fuel [18]. The used relationship has the form:
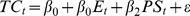
where *TC_t_* is the total cost sustained by the fleet for fuel at year *t*, *E_t_* is the mean cost of fuel at year *t*, and *S_t_* is the yearly cumulative score described above. The model has been estimated for the period 2006–2011.

Finally, revenues and costs associated to each effort pattern are compared in order to compute Gains for year 2011 (***G_2011_***) [58].

### Exploring different scenarios of fishing effort

**Figure 3:**
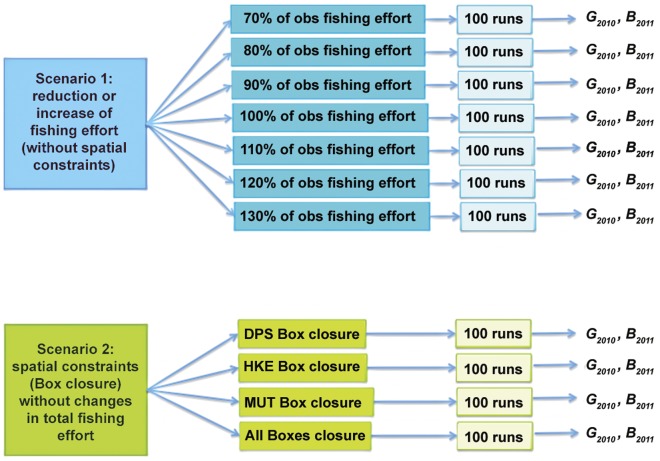
Conceptual scheme of the simulation approach used in exploring different scenarios of fishing effort (Section 3.7).

Classical economic theory predicts that the distribution of fishing effort is determined by the expected economic return to individual fishermen from fishing alternative target species in different fishing grounds [1], [59]–[62]. To preliminarily explore the performance of different management strategies, two types of alternative scenarios of fishing effort pattern in 2010 were simulated and evaluated with respect to corresponding gains and short-time effects on resources abundance (Fig. 3). In the first scenario, the total effort deployed in the GSA16 was modified (reduced or augmented) without any spatial constraint. Seven levels of total fishing effort were analyzed, corresponding to −30%, −20%, −10%, status quo (0% variation), +10%, +20% and +30%, respectively, of the total number of fishing points observed in the real pattern of year 2010 (***Tf_2010_***). In the second scenario, the fishing effort was set to zero in three “boxes” overlapping the nursery areas of the three species, as identified in [63] (Fig. 1d), and the amount of fishing effort originally present in these areas was redistributed outside them. Boxes were defined supposing that the closure of strategic zones (i.e. Marine Protected Areas - MPA), which are fractions of the environment characterized by the presence of essential habitats and/or by the highest presence of resources biomass, can increase resources abundance [64]–[67]. Four situations were analyzed in this scenario, corresponding to the single closure (fishing effort set to zero) of each box (one at a time), and to the simultaneous closure of the three boxes (Fig. 1d).

In the first scenario, it is assumed that a manager controls total fishing effort (by limiting the number of fishermen, days fished, or by other means) but not its spatial distribution. This rationale is valid also for the second scenario, but in this case some areas are excluded by fishing effort allocation.

A number of 100 runs were performed for each level/effort configuration within each scenario (Fig. 3). At each run, the simulation started from the observed resource distribution and the pattern of fishing effort (***Tf_2010_***) obtained modifying the observed one according to the different scenarios. In practice, we counted the number of fishing points in each grid cell to deduce the probabilities to observe points in the different cells. More formally, at each iteration, the location of each single fishing point was modeled as a multinomial distribution with ***Tf_2010_*** trials and support provided by the grid of cells, each having a probability ***p_c_***. The (estimated) probabilities ***p_c_*** of this distribution were obtained from the observed frequencies. The two different types of scenarios were obtained by performing: 1) a variation of the total number of fishing points (***Tf_2010_***) keeping the probabilities ***p_c_*** constant, for the first scenario; 2) a transformation of the probabilities ***p_c_*** keeping the total number of fishing points ***Tf_2010_*** constant, for the second scenario.

In the latter case, we have that:


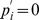
 for cells belonging to nurseries areas or boxes


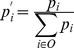
 for cells NOT belonging to nurseries areas or boxes

Where *O* is the set of cells not belonging to the closed box. The simulation proceeded by evaluating at each iteration the new pattern of fishing effort with respect to the associated gains value (***G_2010_***): if this was greater than those obtained at the previous step, the newly generated pattern was accepted and memorized, while the corresponding value of ***G_2010_*** was updated. In this way, the iteration approach was devised to use the ***G_2010_*** as the critical value to be optimized within each run. The run ended when none of a set of 100 newly generated patterns showed a value of *G* greater than the last accepted one. Finally, the corresponding effects of the final fishing effort configuration on the resources abundances for the successive year (2011) were evaluate as follows: the trained EMPNs were used to predict the distribution and abundances by species at year 2011, and the total Biomass (***B_s,2011_***) at year 2011 was used as specific indicator to summarize the stock characteristics for each species. Simulation outputs from both scenarios were evaluated and compared using the respective distributions of ***G_2010_*** and ***B_s,2011_*** as indicators of short-term economic profitability and effect on the stocks, respectively. These indicators were successfully used in bio-economic models based on a simulation approach to inspect and compare outputs [5], [54]. The *status quo* situation, corresponding to a non-spatial management, was used as a baseline.

The overall specific fishing mortality (*F_s_*) was also computed for each simulation output by comparing catches and resources abundances for each size class in number, in order to further inspect the effects of each management approach. Finally, the characteristics of the optimized pattern of fishing effort for each scenario were inspected in terms of a key aspect: the mean distance from coast, which is expected to have a direct impact on costs and then on gains. This was done by computing the Pattern Score (*PS_2010_*), as previously described (see section 3.6).

## Results

### Distribution and abundance of demersal resources

**Figure 4:**
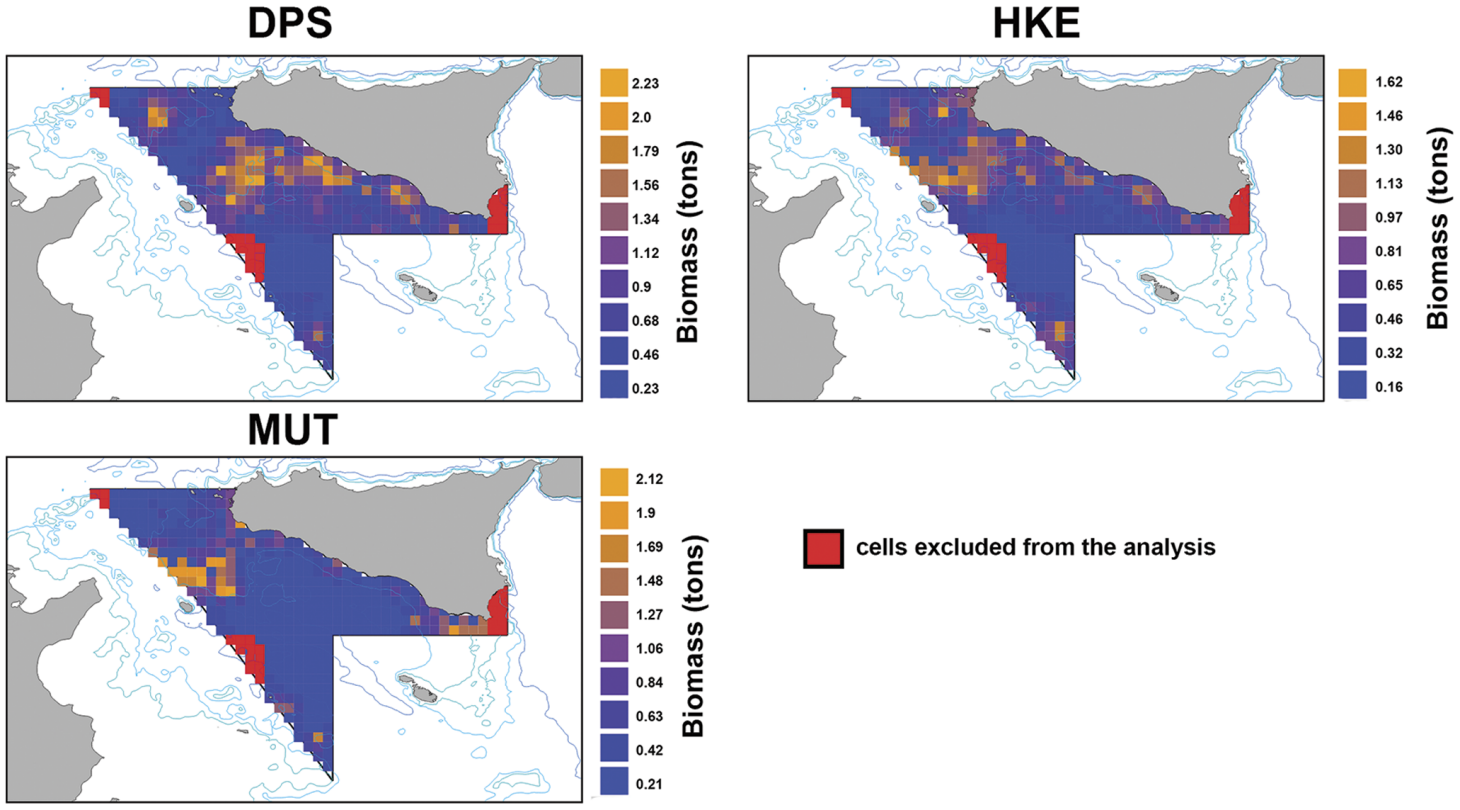
Spatial distribution, for each species/year, of the reconstructed total biomass (Log10 of tons, mean values for the period 2006–2010) obtained after the interpolation procedure of the MEDITS data.

The original multimodal LFDs and the corresponding normal components are reported in Table 3 and visualized, for each species, in Figures S1, S2, S3 to illustrate the process of mixture splitting. In all cases, the K–S tests indicated a high degree of correspondence between raw length distributions and fitted mixtures (data not shown). In the meantime, the characteristics in terms of mean and standard deviation seem to be remarkably stable throughout the years (Table 3). In general, most of the whole DPS biomass is concentrated on the Adventure bank, together with a substantial presence of HKE and MUT (Fig. 4). However, HKE and MUT are mainly located on offshore banks but also on the south of the SoS.

**Table 3:** Values of mean length and standard deviation (sd) by species/cohort/year.

Species	Year	Female (♀) mean length (cm) ± sd			Male (♂)mean length (cm) ± sd		
		Cohort 1	Cohort 2	Cohort 3	Cohort 1	Cohort 2	Cohort 3
***P. longirostris***	**2006**	14.1±2.1	23.4±1.8	26.7±3.5	15.7±1.8	21.0±1.4	25.5±1.6
	**2007**	15.6±2.4	25.0±2.2	31.5±1.9	15.7±1.8	21.0±1.4	25.5±1.6
	**2008**	19.9±2.1	24.4±1.6	28.8±2.5	16.7±1.8	20.5±1.4	25.0±1.6
	**2009**	19.3±3.0	23.6±1.6	27.9±2.5	15.7±1.8	21.0±1.4	25.5±1.6
	**2010**	15.5±3.3	22.8±2.0	30.9±2.1	15.7±1.8	21.0±1.4	25.5±1.6
***M.merluccius***	**2006**	100.0±16.0	200.0±44.8	320.0±110.5	100.0±19.0	190.0±44.2	280.0±59.3
	**2007**	105.0±16.0	200.0±44.8	320.0±110.5	105.0±19.0	190.0±44.2	280.0±59.3
	**2008**	100.0±16.0	200.0±31.3	320.0±110.5	100.0±19.0	190.0±30.9	280.0±59.3
	**2009**	100.0±16.0	200.0±31.3	320.0±110.5	100.0±19.0	190.0±30.9	280.0±59.3
	**2010**	120.0±16.0	220.0±31.3	340.0±110.5	120.0±19.0	210.0±30.9	300.0±59.3
***M.barbatus***	**2006**	144.0±10.0	169.0±21.0	**/**	130.9±7.0	151.0±14.0	**/**
	**2007**	165.0±15.0	176.0±20.0	**/**	123.0±7.0	150.0±14.0	**/**
	**2008**	149.0±12.0	175.0±20.0	**/**	134.0±9.0	154.0±14.0	**/**
	**2009**	159.6±17.0	187.0±19.0	**/**	122.0±8.0	155.0±13.0	**/**
	**2010**	144.0±10.0	169.0±21.0	**/**	134.2±8.0	155.0±13.0	**/**

### Spatial distribution and intensity of fishing effort

**Figure 5:**
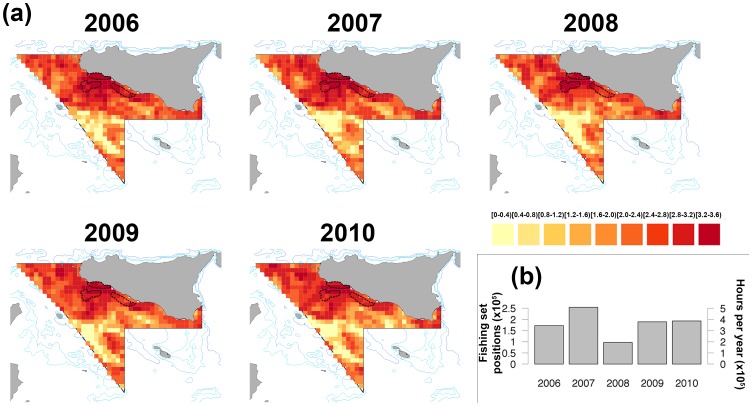
Distributions of the fishing effort in years 2006–2010 (a). Each map represents the 6 min×6 min grid in which the Log10 of the number of fishing points (VMS frequency = 10 minutes) is reported in a yellow-red scale color; b) trend in total fishing effort, from 2006 to 2010, in the GSA 16 area.

Fishing effort appears to be concentrated along the western coast in front of Sciacca and Porto Empedocle and the eastern side of the Adventure Bank (Fig. 5a), where the nurseries of the three species are located (Fig. 1d). Although a high degree of stability of the overall pattern is evident, changes in effort level can be observed in different areas and/or for certain years. Overall, the total fishing effort decreased reaching a maximum in 2007 and a minimum in 2008, while remaining stable in 2009–2010 (Fig. 5b).

### Predicting resources abundances through the artificial neural network

**Figure 6:**
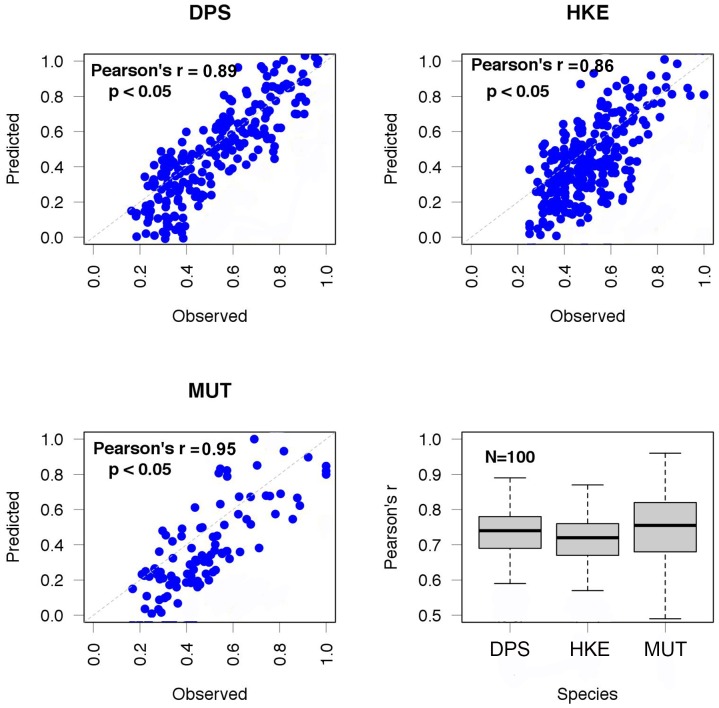
Analysis of trained EMPNs by comparison between observed and predicted abundances for the three species. Each dot represents the average value for a cell, while abundances are reported as relative values (range between 0 and 1). The comparison was carried out on the test subset of data, that is a group of observations (cells) not used during the training phase. Training and testing of EMPN was performed 100 times for each species, since performance could be theoretically influenced by the composition of training and validation dataset, which is randomly determined.

The trained EMPNs for the three species returned a high level of correlation between observed and predicted number of individuals of each cohort. The patterns in Fig. 6a show linear relationships between standardized prediction and observation. The bootstrap procedure applied to evaluate EMPN performance (100 times for each species) returned similar distributions for DPS and HKE (over 80% of correct predictions) and higher for MUT (around 95% - Fig. 6b).

In this way, the data used to feed the EMPNs provided suitable information to forecast the abundances for year 2011. The three EMPNs used in the following analyses were selected within the trained EMPNs with performance close to the distribution means.

**Figure 7:**
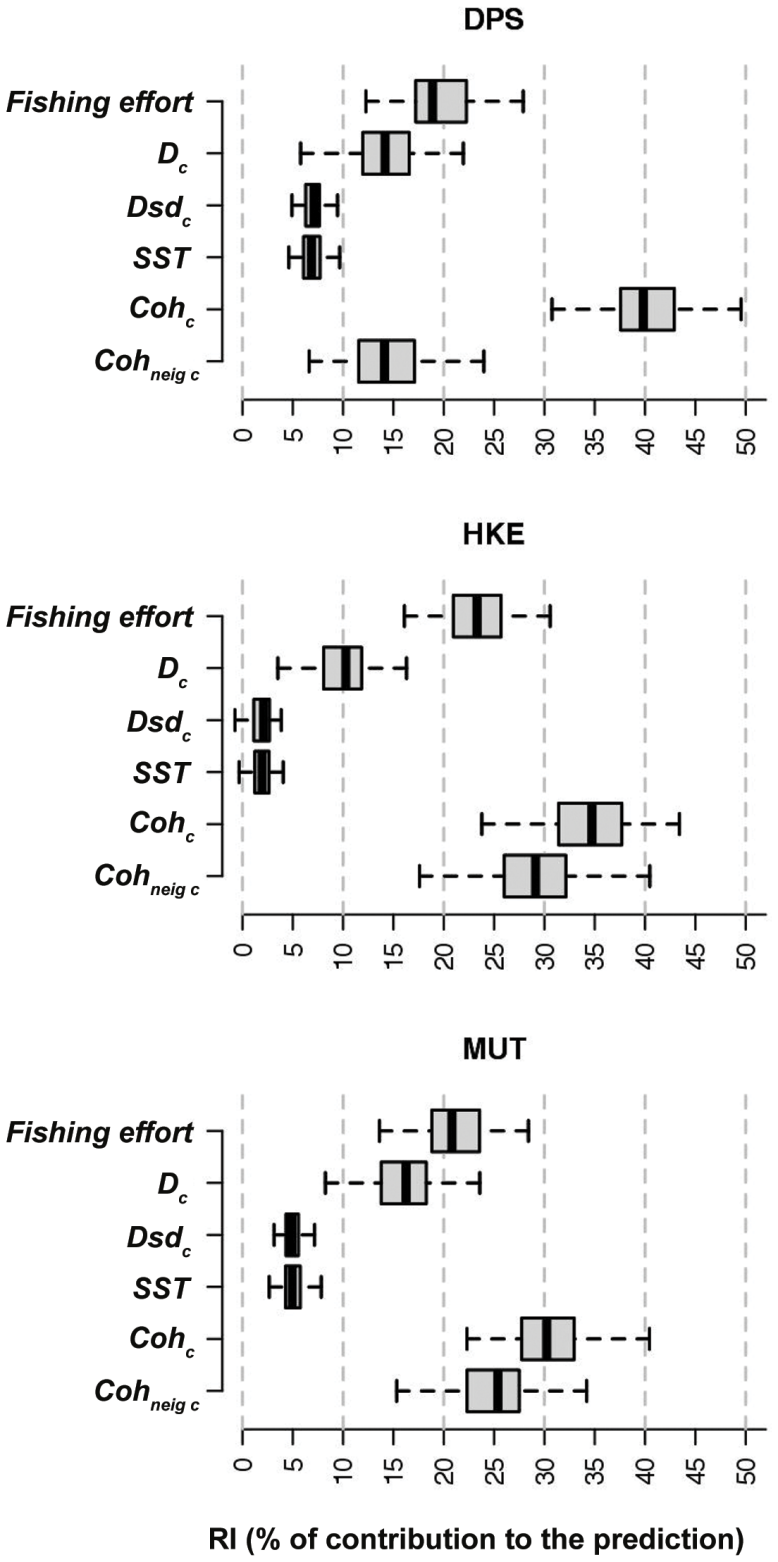
Boxplots of Relative importance index (RI) for the EMPNs input variables, organized in 6 groups for the sake of conciseness and clearness.

The sensitivity analysis showed that the group of input variables characterized by the largest RI, for all the three species, is that corresponding to the cohort abundances for previous years in the same cell (Fig. 7). For DPS, the fishing effort is the secondary factor, while for HKE and MUT the group of abundances in the neighbouring cells precedes fishing effort in terms of RI. In all cases, the RI for 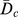 scored around 10%, while RI for *Dsd_c_* and SST ranged between 2 and 7%. Fishing effort is always a not marginal factor.

### Bio-economic model for gains and biomasses

Biomasses, Catches and Total Fishing Mortality computed by SMART were very similar to the values estimated by other models (Table S1). The costs model reported a significant effect of both fuel price and fishing effort pattern (in terms of distance from the coast). The estimated coefficients are reported in Table 4, while a graphical representation of the regression plane can be found in Fig. S4. Both *PS_t_* and *E_t_* positively affect the total annual fuel cost, the second factor being the most important.

**Table 4:** Results of the regression for the costs model (* marks statistically significant values).

Parameter	Estimate	Std. Error	t value	Pr(>|t|)
*Intercept*	−1.607×10^7^	4.933×10^6^	−3.257	0.047*
*PS_t_* (yearly cumulative score)	2.200×10^2^	3.254×10^1^	6.760	0.001*
*E_y_* (mean cost of fuel at year *y*)	1.321×10^7^	3.161×10^6^	4.178	0.025*

**Figure 8:**
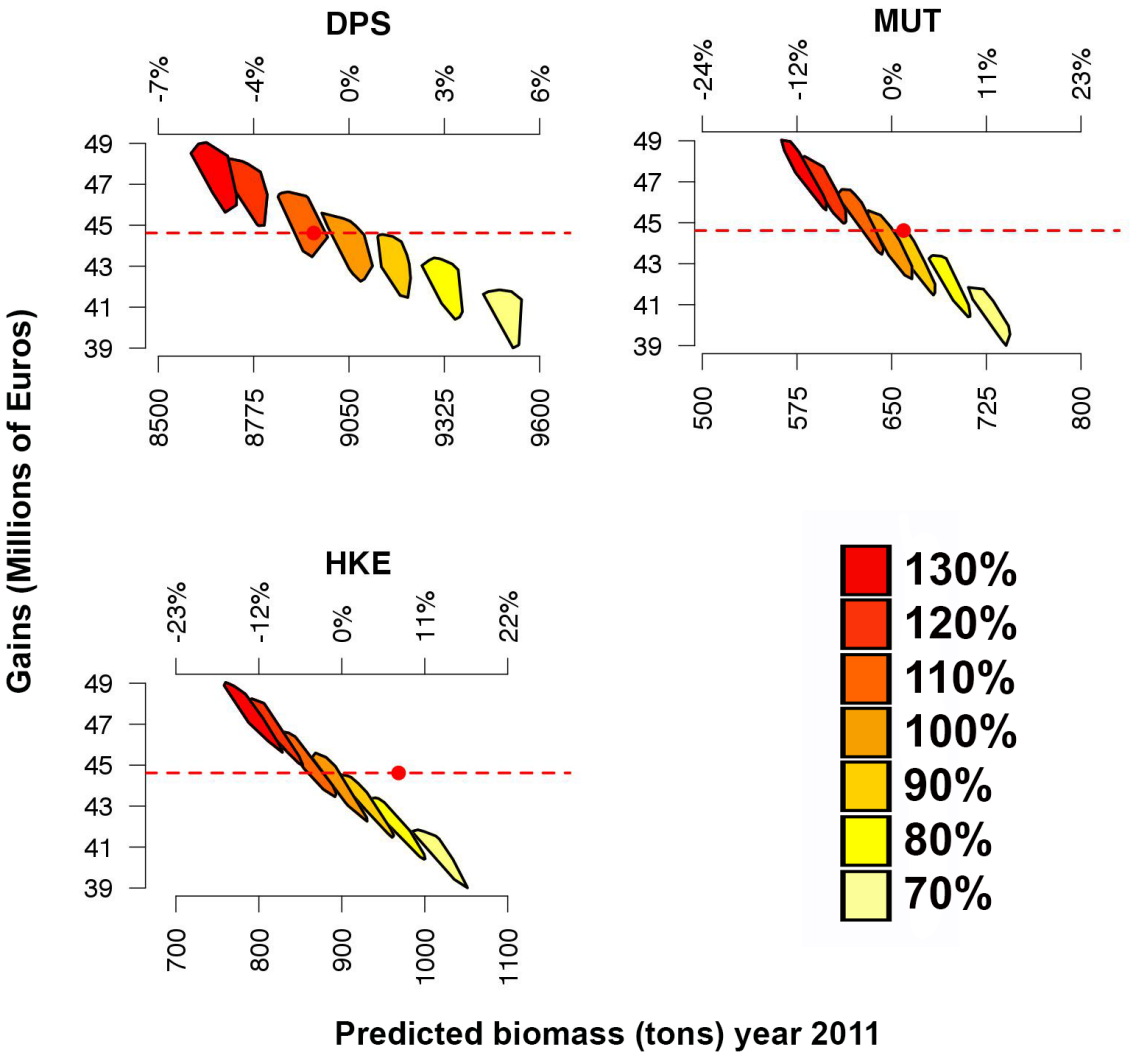
Outputs of the simulations for the first scenario (change of total fishing effort without spatial constraints). Convex hulls are used to represent the set of values of *B_2011_* (Predicted biomass at year 2011) versus *G_2010_* (Gains for year 2010) corresponding to the optimized patterns returned by each run, with red dashed lines and red points represent *G_2010_* and *B_2011_* for the real (observed) pattern of fishing effort in 2010. The bottom x axis reports the absolute values for *B_2011_*, while the top x axis shows the same values as differential percentage from the reference value (*B_2011_* for the observed pattern of fishing effort at year 2010). A yellow-red color scale is used to emphasize the progressive increase of total effort from 70% to 130% of the real (observed) value at year 2010.

The simulation of the first scenarios returns a monotonic relationship between the total effort and economic performance (*G_2010_* of the whole fleet) (Fig. 8). Clearly, *B_2011_* is inversely proportional to the total effort for all three species, whereas gains are linearly proportional to the amount of effort. At one extreme, the reduction of fishing effort to 70% of the value observed in 2010 leads to an increase of *B_2011_* equal to about 5% for DPS, 15% for HKE and 11% for MUT, respectively. Conversely, an increase in fishing effort to 130% of the value observed in 2010 leads to a decrease of about 6%, 13% and 12% of *B_2011_* for DPS, HKE and MUT, respectively. In general, HKE and MUT seem to be the species most affected by changes of effort level, whereas DPS is less sensitive. It is significant that the simulations corresponding to a level of effort equal to that observed in 2010 (that is 100%, without reduction or increase) returned gains values very close to that estimated on the basis of real data (IREPA source). However, the range 70–130% of the total effort corresponds to a range of 40–50 millions of euros for *G_2010_*.

**Figure 9:**
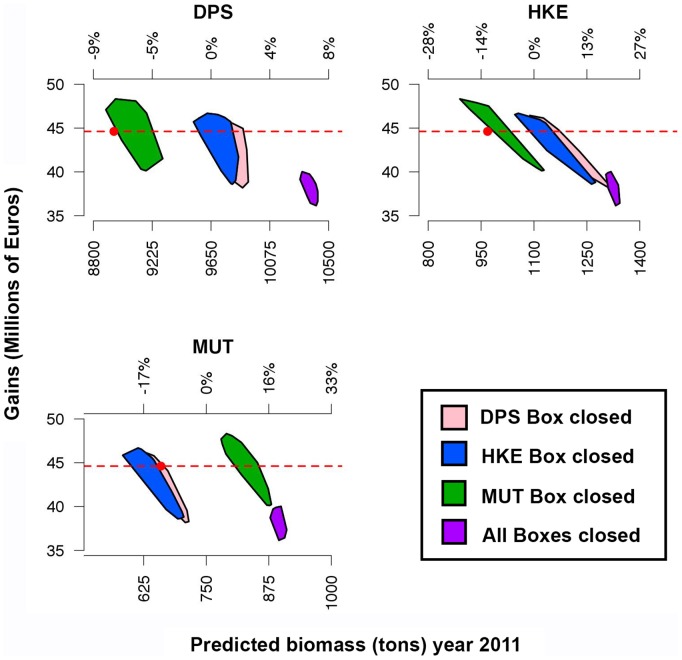
Outputs of the simulations for the second scenario (closure of single or multiple boxes). See caption of Fig. 8 for details.

The closure of single nursery areas (Fig. 9) leads to a benefit, in terms of *B_2011_*, for all species. It is worth noting that some significant synergic effects occur: the closure of DPS nurseries negatively affects MUT but positively affects HKE, whereas the HKE closure has small positive effects on DPS and negative effects on MUT. MUT closure negatively affects the other two species. In all the cases of single box closure, the gains (*G_2010_*) tend to decrease. Conversely, while the simultaneous closure of the three boxes strongly benefits all three species in terms of *B_2011_*, it determines a decrease of gains to a value smaller than that observed for the 70% of total effort (Fig. 8).

**Figure 10:**
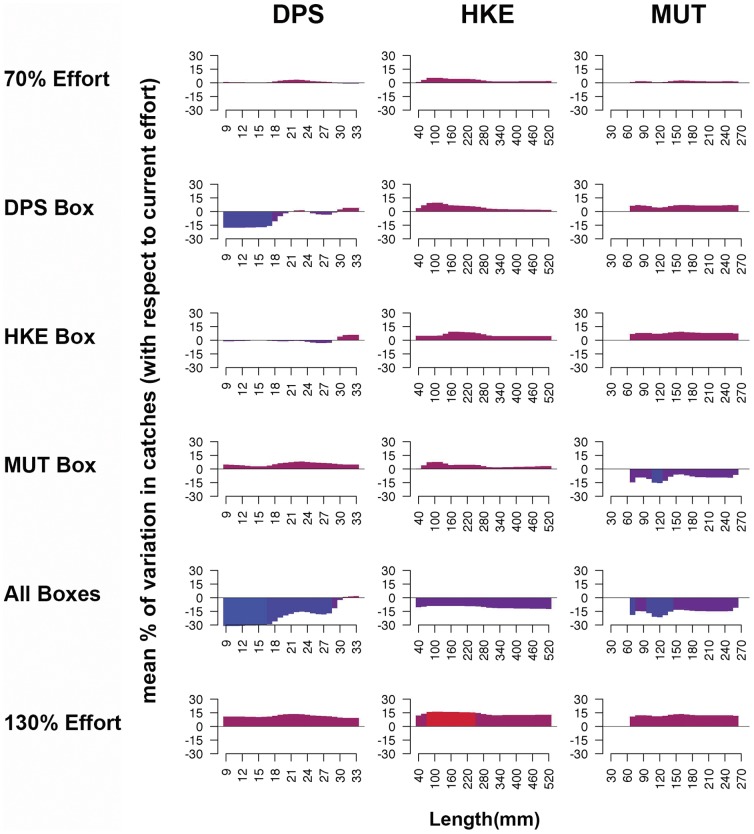
Histograms of the percentage differences between the length-frequency pattern of catches for the simulated pattern of fishing effort and those estimated for the real pattern observed in 2010. Simulations for the second scenario are reported together with the extremes (70% and 130% of the first scenario) as reference for the comparison. Length-classes characterized by a relative increase of catch are highlighted in red, while those characterized by a decrease are highlighted in blue.

In terms of structure of catches obtained for the first scenario, the patterns remain substantially stable among length classes (Fig. 10). Changes in total value of fishing effort determine coherent changes in the total catches, but changes in catches per length class (increase or decrease with respect to total effort) occur over the entire range of lengths. Conversely, in the second scenario, closure of DPS Box returned important changes in the length structure of catches for DPS (Fig. 10), corresponding to a reduction of smallest length classes (lower than 20 mm carapace length). In the case of HKE, the closure of the corresponding Box does not alter the catches profile, whereas closure of MUT Box determines different decrease of catches for different length classes. The simultaneous closure of all the three Boxes produced remarkable effects for DPS and MUT, and particularly for smallest length classes, but have also positive effects for HKE.

**Figure 11:**
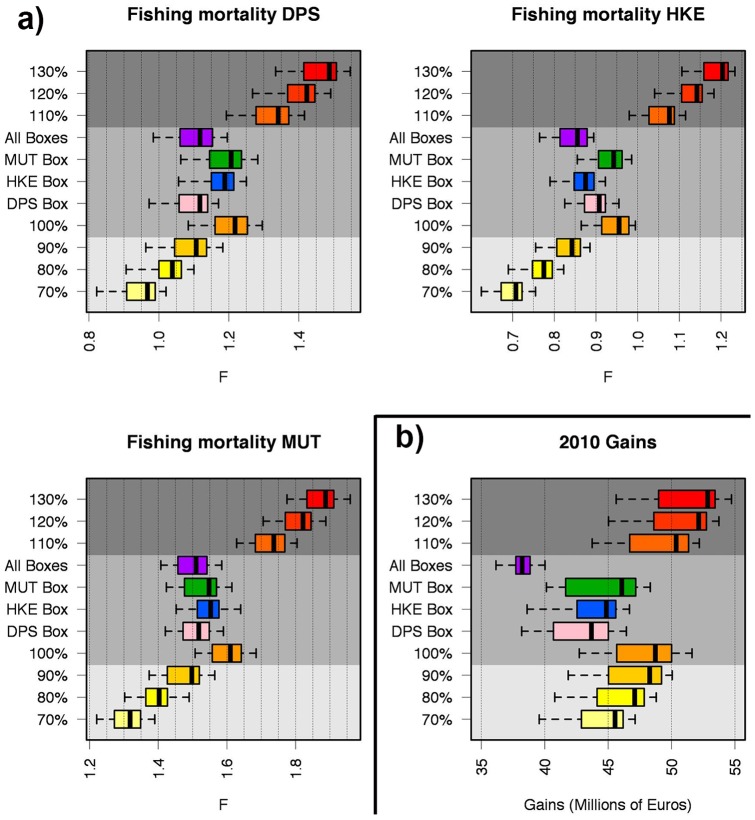
Box plots representing the sets of values for *F_s_* (total fishing mortality for a single species) as function of the applied fishing effort pattern (a). Simulations for the second scenario are located near the 100% effort level, in agreement with the scale of the first scenario. Boxplots of gains (*G_2010_*) corresponding to each simulated pattern (b).

Looking at the total fishing mortalities for the three species (Fig. 11), it seems that: 1) changes of the total allocated fishing effort lead to significant changes in mortalities: 2) the closure of single Boxes (second scenario) implies a reduction of mortality for the related species that is comparable with the effect of a reduction of the about 10% in the total fishing effort (first scenario).

**Figure 12:**
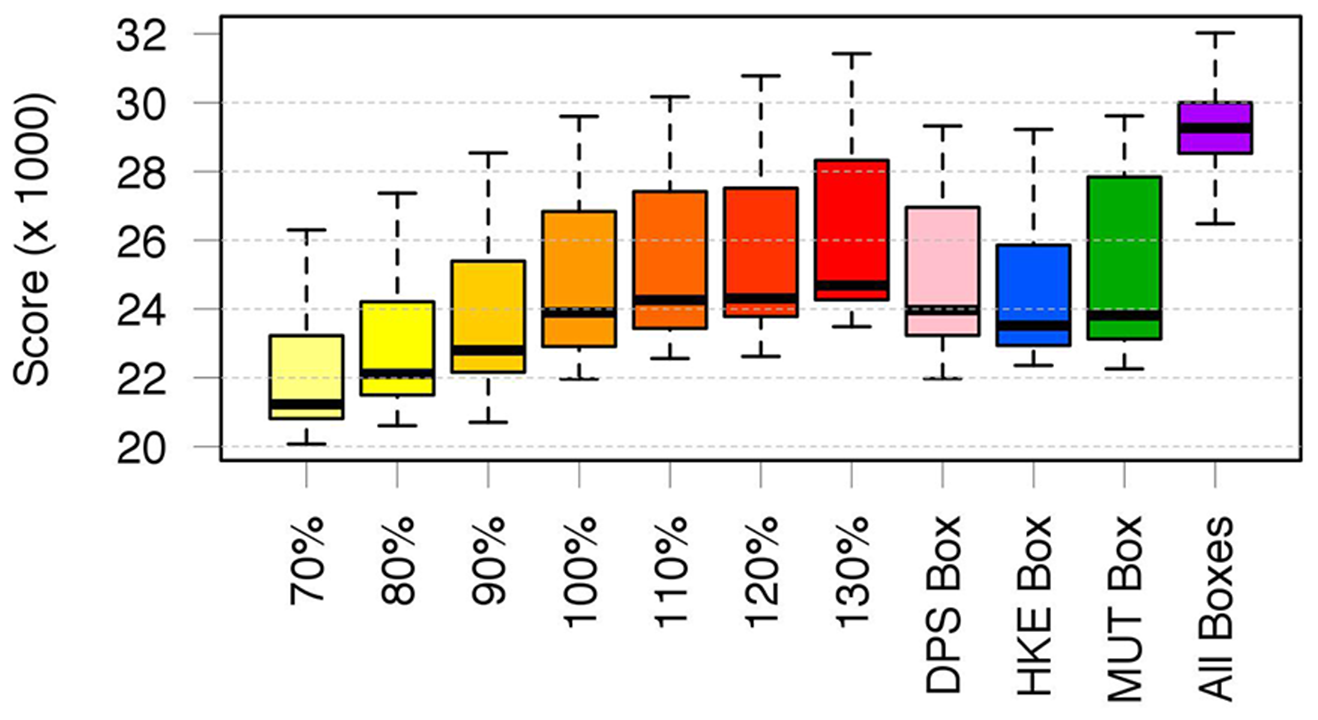
Outputs of the simulations for both scenarios: box plots are used to represent the set of values of Pattern Score (*PS_2010_*), that is the proxy for the evaluation of costs associated to each fishing effort pattern, with respect to the effort configuration, as computed by simulations.

These results can be better understood by looking at the mean *PS_2010_* associated to each scenario (Fig. 12). It is evident that *PS_2010_* is not even directly proportional to the amount of total fishing effort (first scenario), but also that configurations belonging to the second scenario are characterized by *PS_2010_* values which correspond to that observed for the 90% effort level in the first scenario. However, the simultaneous closure of all Boxes determines a dramatic increase of *PS_2010_* (and then of costs) that is reasonably at the base of the gains collapse documented for this configuration.

## Discussion

In recent years growing interest has been shown in fisheries management literature in the use of bio-economic models to represent and analyze the short-term (1–2 years) dynamics of fishing effort in response to regulation [69]–[73]. In this general framework, SMART represents a tool to analyze and predict interactions between groundfish resources and bottom trawl fishing effort, since it is also able to capture the spatial dynamics of resources in the environment and the performance of fisheries in terms of catches, revenues, costs and ultimately gains. SMART has been applied to the demersal fisheries in the Strait of Sicily, taking into account three of the most important commercial species exploited in the study area (both in terms of yields and revenues): the deep water rose shrimp, the European hake and the red mullet. With respect to model performance, our results indicate a good coherence and reliability of SMART, with regard both to its predictive component (trained EMPNs which allow resource distribution to be predicted – Fig. 5), to the deterministic module (see Appendix S1) and finally to the simulation module. The substantial agreement between the optimized pattern for the simulated *status quo* of fishing effort pattern and the real data can be considered as a validation of the approach.

The core of SMART is represented by the interaction between fisheries and demersal resources. It is widely recognized that environmental, trophic and exploitation factors are the main elements regulating fluctuations of stock production in space and time [73]–[75]. In extensively exploited coastal areas, resources abundance is often regulated by fishing effort [14]. Bearing in mind these considerations and working on a short temporal scale (i.e. one or two years), the spatial abundance of a given resource in a given area could be predicted using, as input data, some key environmental descriptors, the observed abundances of the resource and the level of exploitation by fishery in the previous year. To implement the model, Artificial Neural Networks (ANN) were chosen for their ability to recognize and learn the complex non-monotonic and non-linear relationships between biotic and abiotic aspects of the marine environment. Due to their flexibility, the ANNs have been used in different contexts dealing with prediction in fishery ecology, from primary production [51] to fishermen's behavior [41], [76], from stock abundance [77] to spatial distribution of demersal fish species [78]. The results of this first SMART application indicated that internal resources dynamics and fishing effort are more important, as predictive factors at these time scales, than the environmental aspects considered (Fig. 7). This in turn implies that the processes affecting resources dynamics (e.g. ecosystem changes or fluctuations of the specific productivity [79], [80]) can significantly influence SMART in terms of predictive performances and outputs. In this way, the application of SMART is possible only if populations monitoring (i.e. through the MEDITS survey) is guarantee on a regular spatial and temporal basis. Moreover, if the temporal scale of SMART is expanded (e.g. to decades), some changes in cohort parameters could arise.

In our model, human activity (fishing effort) is a factor that regulates system dynamics, determining the near future of the whole system itself. However, this model could be further developed in order to take into account changes of fishing effort through feedback from the system, since variations in resource abundance/distribution will reasonably lead to a rearrangement of fishing effort in space. In particular, as species and stock composition of the groundfish community varies in response to environmental variables and harvesting, fishermen would respond by varying fishing practices (e.g. location of fishing grounds) so as to maintain the highest possible rates of economic return on effort [4].

Classically, there are several different ways of managing fishing mortality: i) by varying fishing effort intensity ii) by changing the exploitation pattern, i.e. the catch by size and iii) by a combination of the above two measures [66]. In areas such as the Mediterranean, characterized by small mesh size in the trawl net cod end (the legal minimum size for European Countries is 40 mm square or 50 mm diamond mesh opening [81]), stock productivity and fleet profitability are generally impaired by a combination of high fishing mortality and inadequate exploitation patterns, with a large number of small sized catches. According to [82], a simple reduction in the current fishing mortality without any change in the fishing selectivity will not allow either stock biomass or fisheries yield and revenue to be maximized. The negative effect of the combination of poor exploitation pattern and high fishing mortality on small sized fish has been reported also for ICES areas [83]. The Authors also pointed to the existence of a trade-off between the exploitation rate and how this affects fish size (exploitation pattern). In particular, either a combination of a relatively high exploitation rate with a low relative exploitation of immature fish, or a combination of a high relative exploitation of immature fish with a low exploitation rate, could contribute to improving current stock status in the northeastern Atlantic. This is in line with the classical theory of Beverton and Holt [68] who first showed that optimal fishing mortality in an exploited stock depends on the relative selectivity of the exploitation pattern.

Due to the poor selectivity of Mediterranean trawl fisheries, a combination of seasonal and spatial closures was proposed as a way to improve the current exploitation pattern by postponing the size/age of young fish on first capture [82].

Following this rationale and these trends in resources management, SMART allows disaggregating the standing stocks by critical areas as a function of the presence of a sensitive life history stage (juveniles and spawners). This property allows fishing mortality to be managed on fractions of the whole population by regulating the use of space by fisheries. Thus, the results of SMART allow to evaluate, in the short term, the effectiveness for the stock status of banning nursery areas (or fractions of them), indicating a significant change of exploitation pattern for two species (DPS and MUT) and the increase in standing stock (as total biomass) for the protected species, thus improving the stock's capacity to avoid risk of depletion or collapse (Fig. 8). In fact, analyzing the main changes in the exploitation pattern associated with DPS and MUT Boxes closure, a decrease in catches for smallest length classes can be observed for the target species (Fig. 10). If all Boxes are closed, a positive improvement in the exploitation pattern of all species is also evident (Fig. 9). From an economic point of view, the simulations of the first scenario indicated that the closing of nurseries (with the total level of fishing effort kept constant) determines a reduction of fishing mortality (Fig. 11) but also implies a significant reduction of total gains (around 15%) for the fleet (Fig. 9). This most likely occurs because all the three banned areas are located near to the coast, and banning them determines a shift of fishing effort towards more offshore areas (Fig. 12), with a likelihood increase of fuel costs.

Apart from economic aspects, the simultaneous closure of Boxes reduces the trawling impact on stocks and is effective for the short-term increase *B_2011_* of all three species. In contrast, the closure of the nursery for a single species seems to determine positive effects for that species (less evident for DPS), but it could also determine adverse impacts for the other two species (except for HKE when MUT Box is closed), in agreement with the results obtained in other multispecies fisheries in which a substantial spatial heterogeneity (e.g. overlap) in the distribution of species exists [5], [84]. Moreover, it should be stressed that the relative effects (increase or decrease in *B_2011_*) of the different simulations should probably reverberate through successive years: if for instance all the Boxes are closed, leading to an increase of *B_2011_* between 5–25% for all the three species, then also the catches and the revenues for the successive years should increase (while the costs should remains almost constant), so that the lowering of gains could be at least partially compensated.

SMART is based on some assumptions: (1) the intra-annual variability of the system is not considered, as the MEDITS survey is carried out once per year; (2) the ANN approach does not explicitly consider the effect of variations of fishing mortalities on survival of recruited fish and on the generation of new recruits by spawning stock; (3) inter-specific interactions are ignored.

With reference to point 1, it is important to observe that most exploited demersal populations move between certain preferential habitats at certain stages of their life cycle. For instance, most of the demersal species aggregate in nursery and spawning areas and fishermen take advantage of this behavior, increasing fishing effort when and where the population is aggregated [69], [83]. The annual scale on which SMART currently works does not take into consideration the fact that areas could be critical for times of less than one year. However, from a conservationist point of view, this apparent limit could be interpreted as a precautionary buffer: to the extent that the closed areas might also be associated with a high fishing effort (and catches) for the rest of the year (relative to areas left open), fishing mortality would be reduced immediately from a given level of nominal effort if they were closed all year round [5]. Recent studies support this rationale [85]–[86]. Furthermore, the closures were expected to allow demersal stocks to build up inside their boundaries since these areas, probably “essential habitats”, are also characterized by the highest concentrations of fish. The possibility of considering the intra-annual dynamics of the system is definitely linked to modeling migration and the different use of the habitat and space of the investigated species during the year.

With reference to point 2, in the absence of an appropriate model, it was not possible to include the increase of biomass due to the growth of survivors and the effect of the increase in spawning stock biomass on the recruitment from a given year through the successive years. These limitations, together with the fact that VMS data have been available only since 2006, lead to the awareness that this model is presently appropriate only for short-term predictions and comparative evaluations of fishing effort patterns mainly during the year in which the measures are adopted.

With reference to point 3, inter-specific interaction (i.e. competition, predation, etc.) in modeled species is assumed to be absent. However, the ANN model developed for this study could be further expanded to incorporate inter-specific relationships.

Although the present form of SMART needs an extensive testing in other areas and some of its modules must be integrated and refined, there is a list of possible alternative scenarios that can be explored. Namely, it could be interesting to explore changes in some key parameters, such as those linked to trawl selectivity. The model also allows combinations of changes in total effort and closure of areas to trawling to be simulated and evaluated.

## Conclusions

Spatially based fisheries management is crucial to sustainability but implies conceptual, technical, and implementation challenges [87]–[89]. This study combines environmental, biological, and fisheries data in a modeling approach aimed to explore the impact of different combinations of fishing effort management, including area closure, on stock status, exploitation impact and fisheries performance. In presenting our model, we have used information and management scenarios for the trawl fisheries in the SoS. In its current form the model is limited to short-term forecasts. The inclusion of aspects linked to migrations of target species (according to life phase and season) and then of explicit stock dynamics is necessary to allow long-term simulation. However, the model provides significant insight into the impact of management measures that can prove useful for shifting multi-species demersal fisheries, such as the trawling fisheries in the Strait of Sicily, from the overfishing status to a more sustainable exploitation. The substantial improvement in exploitation pattern by means of single nursery closure with an increase of biomass for the successive year and a relative decrease of gains, is a major output of the SMART model. According to our results, a series of strategically designed areas of fishery bans could significantly improve trawl fisheries resource conditions in the Strait of Sicily.

## Supporting Information

Appendix S1 **Details about EMPN training.**
(DOCX)

Figure S1 **Histograms of the LFD for DPS males and females (years 2006–2010), with the singles and cumulates probability density functions of the identified cohorts represented by lines.**
(TIF)

Figure S2 **Histograms of the LFD for HKE males and females (years 2006–2010), with the singles and cumulates probability density functions of the identified cohorts represented by lines.**
(TIF)

Figure S3 **Histograms of the LFD for MUT males and females (years 2006–2010), with the singles and cumulates probability density functions of the identified cohorts represented by lines.**
(TIF)

Figure S4 **Representation of the regression between mean annual Fuel price, annual Score (the proxy for fishing effort distance from coast) and total annul expenditure for fuel.**
(TIF)

Table S1 **Comparison between the reference and estimated (by SMART) parameters for some key bio-economic quantities.**
(DOCX)
