# Supplementary material for: SMART: A Spatially Explicit Bio-Economic Model for Assessing and Managing Demersal Fisheries, with an Application to Italian Trawlers in the Strait of Sicily
Source: PLoS One. 2014 Jan 23;9(1):e86222. doi: 10.1371/journal.pone.0086222 (PMC3900514; doi:10.1371/journal.pone.0086222)
Supplement: Appendix S1 — Details about EMPN training. (DOCX) [file pone.0086222.s001.docx]

**Appendix S1.** **Details about EMPN training**

The application of an MPN or EMPN consisted of a training phase and a test phase. The training is based on the use of two datasets (the “training” and “validation” datasets, respectively), which were used to adjust the weights of the hidden layer neurons, that is to minimize the error function (generally the mean squared error – MSE) between the observed and predicted values. In the test phase, the performance of EMPN in classifying patterns from a “test dataset”, which is different from both the training and the validation dataset, is assessed. The use of two datasets during training is justified by the fact that, in this way, the risk of “overfitting” is drastically reduced. Overfitting occurs when an EMPN adjusts to the training dataset so well that its ability to generalize and correctly classify new patterns is partially lost. Given that overfitting occurs when the MSE for the training dataset continues to become smaller, while that for the validation dataset begins to increase, the occurrence of overfitting can be avoided using an early-stopping strategy: when a systematic increase in the error for the validation dataset occurs, the training is stopped [47]. In order to adopt this approach, the 394 cells of the grid were randomly grouped into three complementary (and thus not overlapping) subsets of size 50, 25, and 25%, respectively. The relative sizes of the training, validation and test subsets were set at reasonable values [45] since the literature is not conclusive regarding an “ideal” size for each partition [48-50]. A similar criterion was used to set the number of neurons in the hidden layer: it was set as double the number of input variables [51]. The datasets thus obtained were used to feed and test the EMPN: random regrouping of dataset and relative training and test were performed 100 times, since EMPN performance are at least partially affected by the composition of training datasets.
