# Supplementary figures and images for: SMART: A Spatially Explicit Bio-Economic Model for Assessing and Managing Demersal Fisheries, with an Application to Italian Trawlers in the Strait of Sicily
Source: PLoS One. 2014 Jan 23;9(1):e86222. doi: 10.1371/journal.pone.0086222 (PMC3900514; doi:10.1371/journal.pone.0086222)

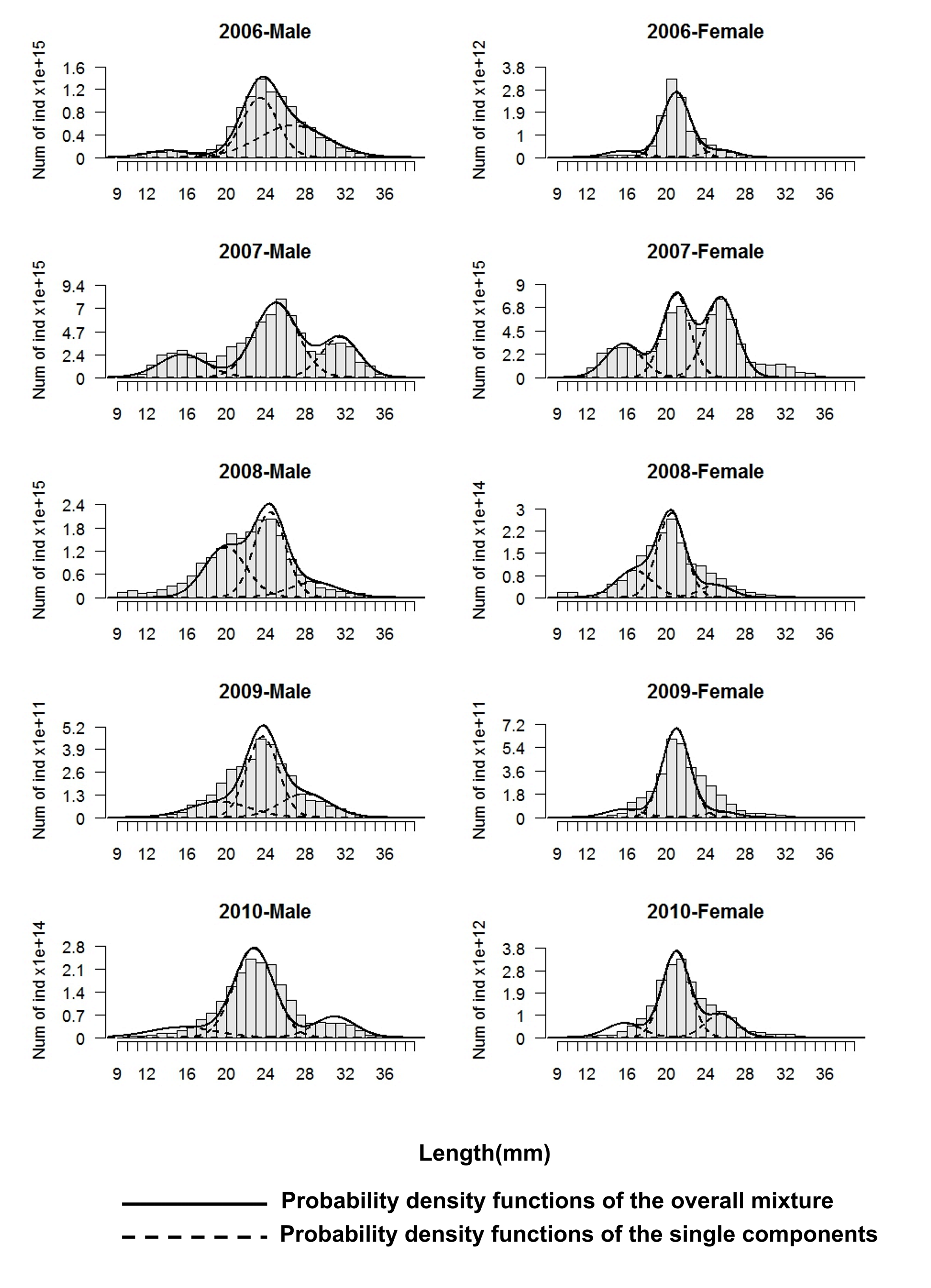

Supplement: Figure S1 — Histograms of the LFD for DPS males and females (years 2006–2010), with the singles and cumulates probability density functions of the identified cohorts represented by lines. (TIF) [file pone.0086222.s002.tif]

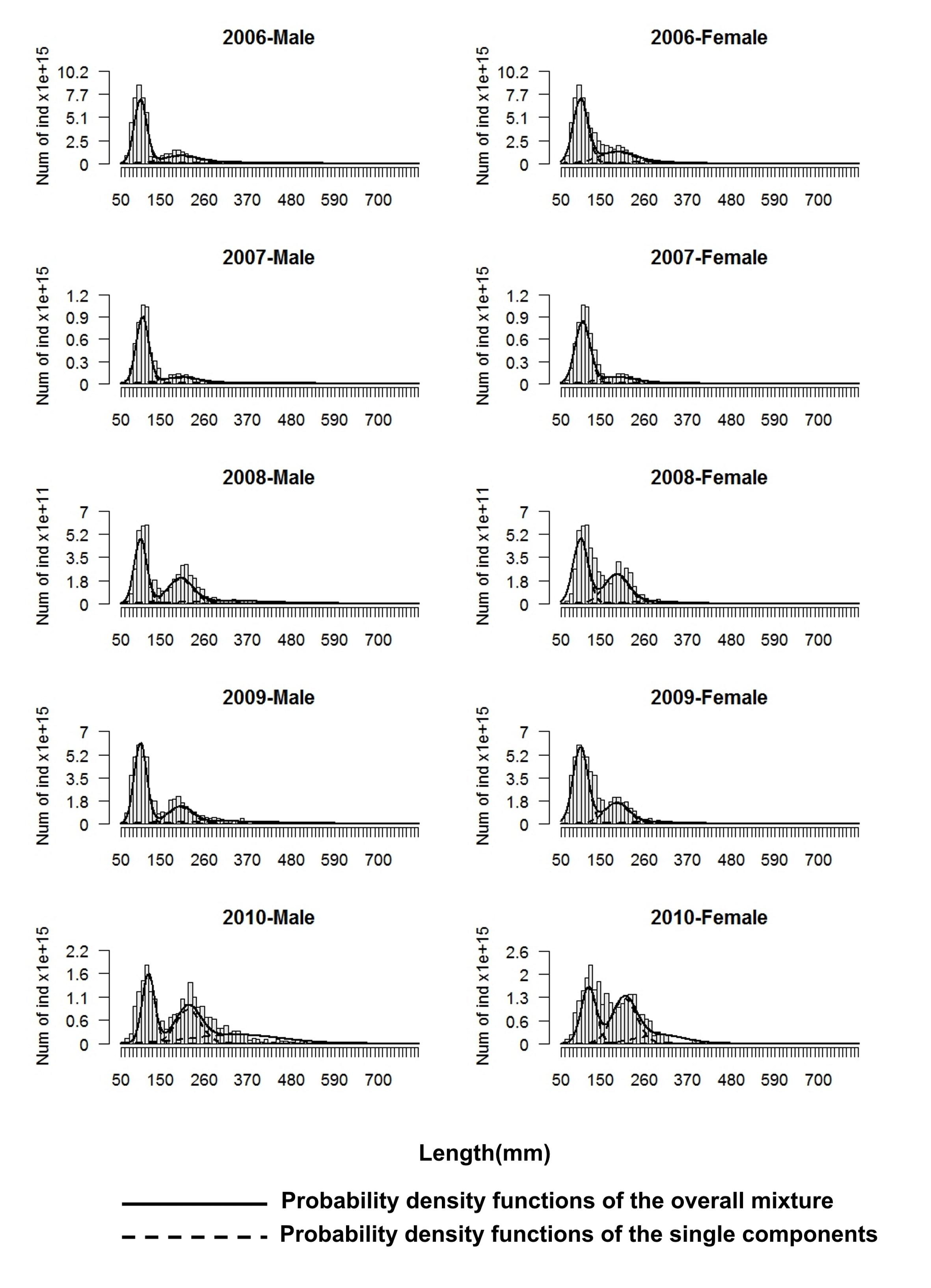

Supplement: Figure S2 — Histograms of the LFD for HKE males and females (years 2006–2010), with the singles and cumulates probability density functions of the identified cohorts represented by lines. (TIF) [file pone.0086222.s003.tif]

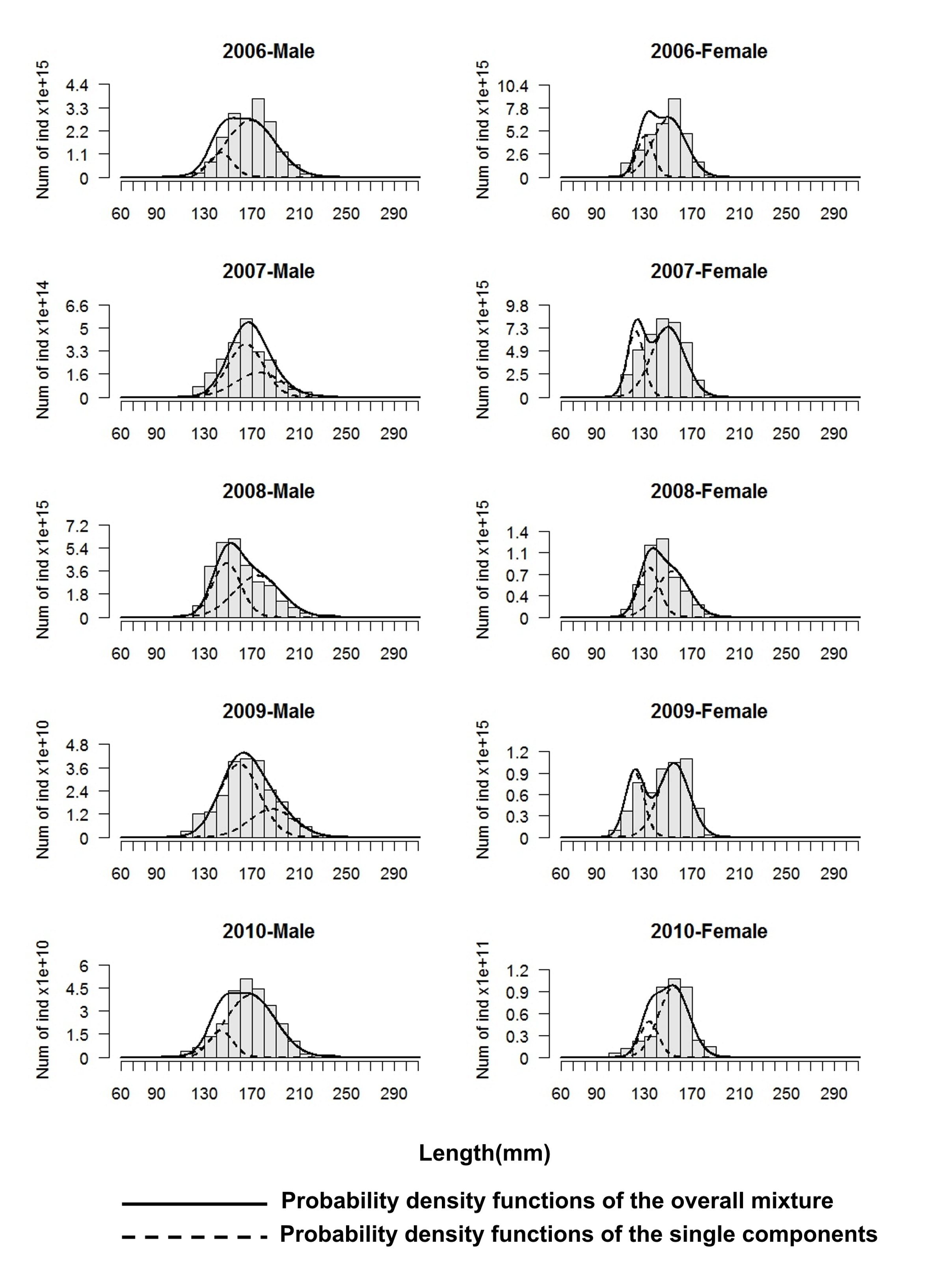

Supplement: Figure S3 — Histograms of the LFD for MUT males and females (years 2006–2010), with the singles and cumulates probability density functions of the identified cohorts represented by lines. (TIF) [file pone.0086222.s004.tif]

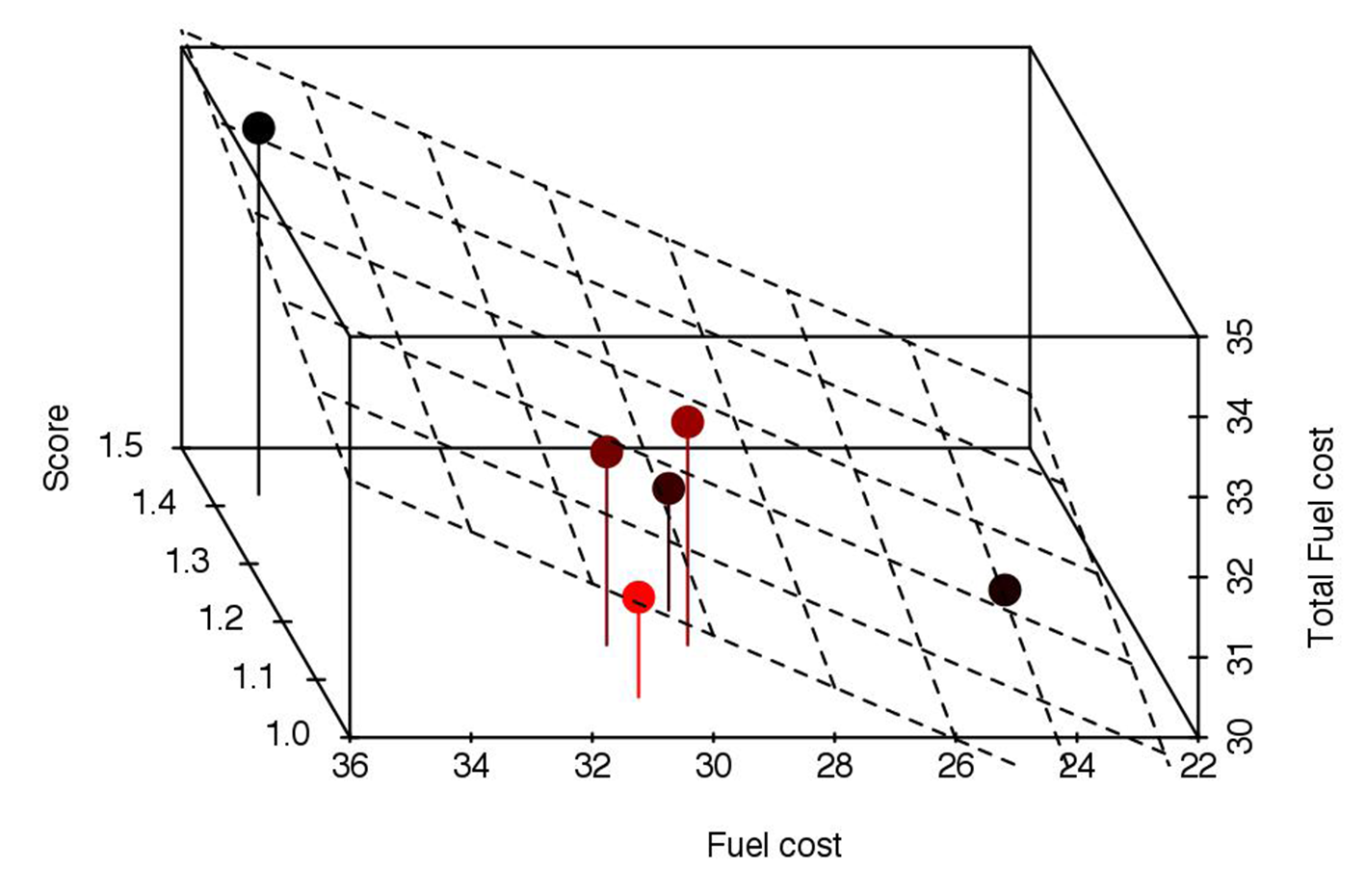

Supplement: Figure S4 — Representation of the regression between mean annual Fuel price, annual Score (the proxy for fishing effort distance from coast) and total annul expenditure for fuel. (TIF) [file pone.0086222.s005.tif]
