# Supplementary material for: SMART: A Spatially Explicit Bio-Economic Model for Assessing and Managing Demersal Fisheries, with an Application to Italian Trawlers in the Strait of Sicily
Source: PLoS One. 2014 Jan 23;9(1):e86222. doi: 10.1371/journal.pone.0086222 (PMC3900514; doi:10.1371/journal.pone.0086222)
Supplement: Table S1 — Comparison between the reference and estimated (by SMART) parameters for some key bio-economic quantities. (DOCX) [file pone.0086222.s006.docx]

|  | Species | | | | | |
| --- | --- | --- | --- | --- | --- | --- |
|  | DPS | | HKE | | MUT | |
| Parameter | Reference | SMART estimation | Reference | SMART estimation | Reference | SMART estimation |
| Total Biomass 2010 GSA16 | 4860 | 4928.3 | 1222 | 1221.1 | 611 | 588.5 |
| Total catches 2010 (tons) | 3713.4 | 3964.7 | 733.1 | 727.0 | 427.5 | 436.5 |
| Biomass year 2011 | 9980 | 9225 | unknown | 1076 | unknown | 732 |
| Total fishing mortality (F) from LCA | 1.32 | 1.3 | 1.14 | 1.11 | 1.46 | 1.42 |
